# First report of *MEFV* duplication in FMF patient

**DOI:** 10.1186/1546-0096-13-S1-O15

**Published:** 2015-09-28

**Authors:** G Sarrabay, D Méchin, B Dumont, M André, I Touitou

**Affiliations:** 1 CHU Montpellier, Montpellier, France

## Introduction

Familial mediterranean fever (FMF) is a rare monogenic disease and the prototype of autoinflammatory disorders. It is caused by mutations in the *MEFV* gene and is autosomal recessively inherited. Most mutations are missense substitutions, small deletions are quite rare, and only three nonsense mutation has been described (http://fmf.igh.cnrs.fr/ISSAID/infevers/). Large rearrangements have been searched for in the frame of a collaborative project including 216 patients but were not identified.

## Objectives

We report here the first case of *MEFV* duplication in a FMF patient.

## Patients and methods

The proband is a 21 years-old woman who presented with classical FMF phenotype: recurrent fever, arthralgia, and abdominal pain with vomiting. Attacks lasted three days and biological inflammation was documented with elevated C-reactive protein. Her father is Armenian and her mother Malagasy, and both are asymptomatic. We performed Sanger analysis (ABI3130x, Life Technologies) of the *MEFV* gene, quantitative polymerase chain reaction (qPCR) (LighCycler, Roche) and deep-sequencing (Nextera Rapid Capture, Illumina) (MiSeq, Illumina). Microsatellite analysis (ABI3130x, Life Technologies) was also performed.

## Results

We identified a well-known severe mutation: p.Met694Val, and a controversial variant: p.Glu148Gln. Parental testing confirmed that the variants were non-allelic. Sanger sequencing displayed unbalanced ratio of the mutated and wild type alleles. Mosaicism was excluded because all polymorphisms were found at the same 1:2 ratio. DNA contamination was ruled out through microsatellite analysis. We thus suspected a gene micro-rearrangement. qPCR and deep-sequencing revealed a heterozygous duplication of the entire wild *MEFV* gene. The two surrounding genes (*NAA60* and *OR1F1*) were not duplicated demonstrating that this rearrangement was confined to the *MEFV* region. qPCR analysis showed that the duplication was inherited from the mother.

## Conclusion

We report here the first FMF patient with 1/3 dose of p.Met694Val. Interestingly, the patient's phenotype seemed not to be impacted by the “dilution” of the pathological variants.

## Consent to publish

Written informated consent for publication of their clinical details was obtained from the patient/parent/guardian/relative of the patient.

